# Prevalence of obesity among school-age children and adolescents in the Gulf cooperation council (GCC) states: a systematic review

**DOI:** 10.1186/s40608-018-0221-5

**Published:** 2019-01-08

**Authors:** Hanouf Al Hammadi, John Reilly

**Affiliations:** University of Strathclyde, Physical Activity for Health Group, Glasgow, G1 1XQ Scotland

**Keywords:** Children, Adolescents, Obesity, Body mass index, Systematic review, Gulf cooperation council (GCC)

## Abstract

**Background:**

The Gulf Cooperation Council (GCC) countries have among the highest prevalence of adult obesity and type 2 diabetes in the world. This study aimed to estimate the recent prevalence of obesity among school-age children and adolescents in the GCC States.

**Methods:**

The literature search for obesity prevalence data was carried out in July 2017 in Google Scholar, Physical education index, Medline, SCOPUS, WHO, 2007–2017, and updated in November 2018.In addition, 22 experts from the GCC were contacted to check the search results, and to suggest studies or grey literature which had been missed. Eligible studies were assessed for quality by using the Joanna Briggs Institute (JBI) tool for prevalence studies. Conduct of the systematic review followed the Assessment of Multiple Systematic Reviews Tool (AMSTAR) guidance. A narrative synthesis was conducted.

**Results:**

Out of 392 studies identified, 41 full-text reports were screened for eligibility; 11 of which were eligible and so were included, from 3 of the 6 GCC countries (United Arab Emirates, Kuwait, Saudi Arabia). Surveillance seems good in Kuwait in compared to other countries, with one recent national survey of prevalence. Quality of the eligible studies was generally low-moderate according to the JNBI tool: representative samples were rare; participation rates low; power calculations were mentioned by only 3/11 studies and confidence intervals around prevalence estimates provided by only 3/11 eligible studies; none of the studies acknowledged that prevalence estimates were conservative (being based on BMI-for-age). There was generally a very high prevalence of obesity (at least one quarter-one third of study or survey participants obese according to BMI-for-age), prevalence increased with age, and was consistently higher in boys than girls.

**Conclusions:**

The prevalence of obesity among school-age children and adolescents appears to have reached alarming levels in the GCC, but there are a number of major gaps and limitations in obesity surveillance in the GCC states. More national surveys of child and adolescent obesity prevalence are required for the GCC states.

**Trial registration:**

PROSPERO registration number CRD42017073692.

**Electronic supplementary material:**

The online version of this article (10.1186/s40608-018-0221-5) contains supplementary material, which is available to authorized users.

## Background

The Gulf Cooperation council countries include Kuwait, the United Arab Emirates (UAE), Qatar, Bahrain, the Kingdom of Saudi Arabia (KSA), and Oman. The GCC countries have among the highest adult obesity and type 2 diabetes prevalence in the world [1–4] with rapidly increasing prevalence of adult obesity and diabetes in the past two decades [1]. Several factors have contributed to the high prevalence of obesity in the GCC countries [2], notably the very large and rapid increases in household income, with associated lifestyle changes that include reduced physical activity and increased consumption of obesogenic foods and drinks [5, 6].

Surveillance of childhood obesity is considered central to tackling the obesity epidemic [7], but there may be a number of important limitations in surveillance of child and adolescent obesity prevalence in the GCC at present. Specifically, we were unable to find a recent systematic review of obesity prevalence of children and adolescents in the GCC, so prevalence of the problem is unclear. Our initial scoping review also suggested that many previous studies combined the prevalence of overweight and obesity, and so the prevalence of obesity could not be determined. In addition, many previous studies in the GCC collected data over 10 years ago and these studies may now be out of date given the rapid increases in prevalence in the region [8, 9]. The recently published global estimates of obesity prevalence [10, 11] used data from the GCC countries which were also over 10 years old for example, so there is a need for prevalence data from more recent studies and surveys. An additional problem with older evidence is the fact that definitions of child and adolescent obesity have evolved over the past decade. Specifically, the WHO definition of child and adolescent obesity based on BMI-for-age was not published until 2007, and was not in widespread use until some time after that. A further problem with existing obesity prevalence data is that systematic reviews demonstrating limitations of BMI-for-age as a surveillance tool (high specificity for excessive fatness, but only low-moderate sensitivity) have become available only relatively recently [12, 13] and recent obesity prevalence studies or surveys from the GCC countries may not have made allowances for this important source of bias in prevalence estimates.

The primary aim of the present study was therefore to establish the recent (last 11 years) prevalence of obesity among school-age children and adolescents in the GCC states. Secondary aims were: to identify differences in prevalence between countries and between groups (e.g. by gender, age); to identify major gaps and weaknesses in the evidence base on obesity prevalence in the GCC. The study was intended to help improve child and adolescent obesity surveillance in the GCC in future, so that public health action aimed at tackling the obesity epidemic in the GCC can be better informed [7].

## Methods

### Registration and reporting of the systematic review

This systematic literature review was reported in accordance with the Preferred Reporting Items for Systematic Reviews (PRISMA) guidelines [14]. The review protocol was registered on PROSPERO on the 10th September 2017 (registration number CRD42017073692), the international prospective register for systematic reviews (https://www.crd.york.ac.uk/prospero/display_record.php?RecordID=73692). The search strategy followed the PECO (population, exposure, comparator and outcome) format: population = school-age children and adolescents in the GCC countries; exposure = obesity as defined using BMI-for-age; comparator = any appropriate BMI-for-age reference data; outcome = prevalence of obesity among the general population since 2007.

### Literature search

The literature search was originally conducted on 17 July 2017. The manuscript was submitted to this journal on 28th February 2018 and reviews were not received until October 2018, so the original searches were repeated on 2nd November 2018. Searches used the five most relevant electronic databases: Medline, Google Scholar, Physical education Index, SCOPUS and WHO. The search terms used in Medline are provided in Additional file 1: Table S1, as required by the PRISMA checklist; terms were very similar in the other databases, though with small differences in syntax between databases. The electronic database searching was complemented by reference citation tracking (forward and backward) of the included studies and of previous reviews, consultation with GCC-based experts in the field, and a search for ‘grey literature’ among the GCC experts (summarised in Additional file 2: Table S2).

### Study selection

#### Inclusion criteria

Studies were included if they satisfied all of the following inclusion criteria: Prevalence data were collected in the last 11 years, i.e. from Jan 2007 to end October 2018; from a GCC country (Bahrain, Kuwait, Qatar, Oman, KSA, UAE); they provided prevalence of obesity rather than overweight prevalence, or prevalence of overweight/obesity combined; they must have defined obesity using an accepted method for children and adolescents based on BMI-for-age; BMI must have been based on measured height and weight (rather than self-report or parental report); age of study participants between 5 and 19 years; study participants from the general population (e.g. not from clinical samples).

#### Exclusion criteria

Studies were excluded if they addressed prevalence of obesity in middle Eastern countries that are not members of the GCC, with study participants outside the range 5–19; if prevalence data were collected earlier than 2007; if prevalence estimates from BMI-for-age were based on self-reported height or weight, or which combined prevalence of obesity and overweight so that obesity prevalence could not be ascertained. Studies that sampled from specific populations (e.g. clinical populations) were also excluded.

All literature search hits, and all potentially eligible studies identified by forward and backward citation searching, were examined for eligibility independently by both authors. The two authors resolved differences of opinion over eligibility by discussion, and in a few cases, by asking for clarification of methods from the authors of the original studies (e.g. over the precise data collection period where this was unclear in a few cases). A list of studies excluded at the full-text screening stage, with reason(s) for exclusion is providing in the supplementary material (Additional file 3: Table S3).

#### Data extraction

A data extraction form was devised in advance of the process of data extraction and used to populate the evidence tables given in the Results section below, with summary data on prevalence of obesity (with 95% CI where possible) overall, and by subgroup as reported (e.g. by age, gender, definition of obesity). Both authors independently populated the data extraction forms, and they resolved any differences of opinion by discussion.

#### Quality assessment-appraisal of eligible studies

The quality of individual eligible studies was assessed by both authors independently using the Joanna Briggs Institute (JBI) checklist for assessment of the quality of prevalence studies [15]. Two additional aspects of prevalence study quality which are specific to obesity were also considered and added to the data extraction forms: the timing of data collection-given recent rapid increases in obesity prevalence in the GCC; [8, 9]; whether biases arising from use of the BMI-for-age to estimate obesity prevalence [12, 13] had been considered by the authors (e.g. reported in the Discussion and/or used to adjust prevalence estimates in the Results).

#### Guidance on maintaining the quality of the systematic review

In an attempt to ensure high quality of the present review *process* the authors planned and conducted the review by addressing each of the items in the Assessment of multiple systematic reviews tool (AMSTAR [16]) checklist -the process is summarised in Table 1.

**Table 1:** AMSTAR Self-assessment of the process used in the present study

Questions	Yes/NO	Answers in detail
1) Did we have a PICO/PECO	Yes	Population = children and adolescents of school-age as defined by WHO (5-19 yrs) from GCC countries; E = exposure = obesity defined using an acceptable method based on BMI-for-age; Comparator = any appropriate reference data; Outcome = prevalence of obesity rather than overweight and not overweight/obesity combined, from 2007 onwards.
2) Did we specify review methods in advance of doing the review?	Yes	Registered in PROSPERO ref. CRD42017073692
3) Did we explain/justify inclusion criteria (based on study design)?	Yes	We did not exclude any study design, but RCT not likely to be that relevant (though RCT could contain relevant data and so not excluded)
4) Did we have a comprehensive lit search strategy?	Yes	Electronic databases from 2007 to 2018: See Methods and Additional file 1.
		We were resourced to search for English language publications only, though literature suggested by expert contacts in the GCC (including grey literature) in Arabic would have been considered eligible.
		Searched reference lists of eligible studies? Yes, both forward and backwards citation searching was carried out.
		Consulted experts? Yes-list of *n* = 22 expert contacts in all GCC countries was consulted in October–November 2017.The experts from the GCC were asked to check on the results of the literature search and if they were aware of any grey literature which was relevant (e.g. national surveys)
		Search within 24 month of planning the search - Yes, within 2 months
5) Did review authors agree on inclusions/exclusions of full text potentially eligible studies?	Yes	Both authors agreed on inclusions/exclusions of the full text articles screened
6) Data extraction from eligible studies- did both review authors do this/check this?	Yes	Both authors agreed on data extraction from the eligible studies
7) Is a list of all excluded studies provided, including the reasons for exclusion?	Yes	Appendix of excluded studies and reasons for exclusion is provided (Additional file 3).
8) Does the data extraction and narrative (Evidence tables and text) provide enough detail about the eligible studies?	Yes	Text summarises the eligible studies, and details of eligible studies are provided in Evidence Tables (Results section)
9) Did the review authors consider risk of bias?	Yes	Results section (Evidence Tables) contains data on possible sources of bias including: sample size; representativeness of sample; bias arising from definition of obesity used.
10) Did the review authors report sources of funding of their review?	Yes	Kuwait Cultural Office and Scottish Funding Council
11. & 12 Did the review authors do a meta-analysis? (Was this appropriate?)	No	No meta-analysis possible dues to degree of differences in study design and methods: different nations; differences in factors which create differences in prevalence estimates- different definitions of obesity, different age groups, different sex distributions in studies
13) Did the review authors consider sources of bias in eligible studies?	Yes	As above- the evidence tables deal with representativeness, sample size/power calculations, and biases in the definitions of obesity used by the studies
14) Did review authors consider sources of heterogeneity in eligible studies?	Yes	Different times, different obesity definitions, different ages and sexes, different countries and places all considered
15) Did review authors consider other sources of bias (in particular publication bias)	N/A	No formal testing for publication bias was possible due to small number of eligible studies.
		Main sources of bias in prevalence studies were considered: sample size and representativeness; use of BMI to estimate obesity prevalence is biased (underestimates obesity prevalence) as noted in the manuscript.
16) Did review authors consider any conflicts of interest which arose when doing their review?	Yes	No conflicts to declare

#### Synthesis of study findings

A meta-analysis of review findings was considered desirable if practical, but it was recognised that marked gaps in the evidence and /or differences between studies, e.g. differences in age or sex, ethnicity or socio-economic status of the samples, differences in time period [17], differences arising from differences in the definition of obesity used -which would be expected to be marked [12]-might preclude meta-analysis. Publication bias assessment was also considered desirable if possible.

## Results

### Eligible studies and study selection process

**Fig. 1:**
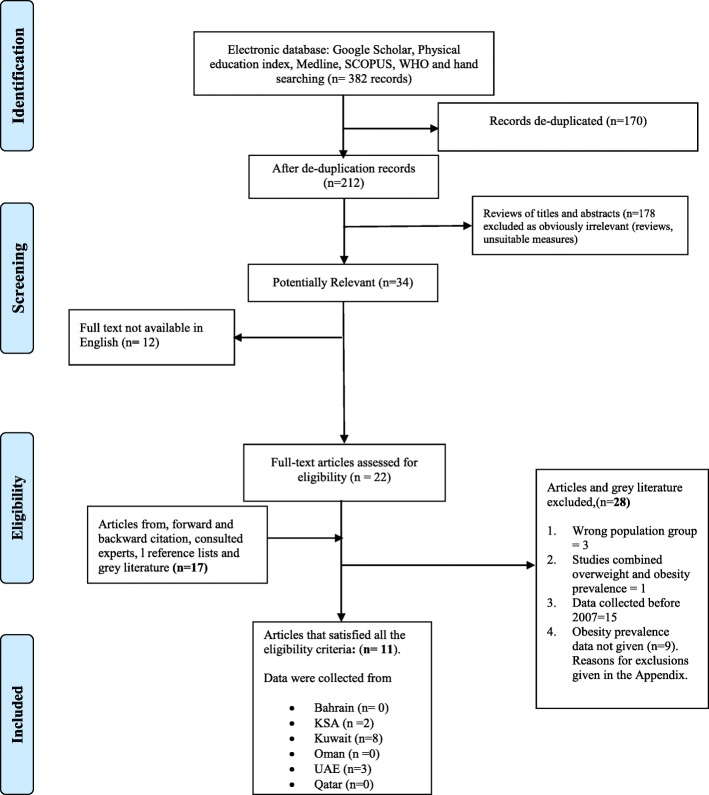
PRISMA Study Flow Diagram. Footnote: search updates in November 2018 identified 10 additional potentially eligible studies, of which 6 were excluded from Abstract screening and four were deemed ineligible after full-text screening (see Additional file 3)

The PRISMA flow diagram is provided in Fig. 1. In the original search twenty-two potentially eligible papers were identified for full-text eligibility screening by both authors by the conventional literature searching and a further 17 papers/surveys from the grey literature were suggested by expert contacts in the GCC and all of those were full-text screened by both authors. The search update in November 2018 identified further studies for full-text screening but no additional eligible studies, so only 11 eligible papers/survey reports were identified, reporting 13 separate prevalence estimates, from 3 of the 6 GCC countries (UAE, Kuwait, KSA): there were no eligible studies from Oman, Bahrain, and Qatar. In summary, the number of eligible studies in the GCC countries was limited and the studies/surveys themselves differed substantially by time, sample age and sex, ethnicity, and by definitions of obesity used. No formal assessment of publication bias was possible given the small number of eligible studies, and so a narrative synthesis, by nation, is provided below.

### Study quality appraisal

The formal appraisal of study quality for the 11 eligible studies/surveys is summarised in Table 2. A number of the quality assessment items were not reported or not carried out, in particular the use of nationally representative samples was rare (1/11 eligible studies), and few studies reported power calculations or confidence intervals for their prevalence estimates (only 3/11 provided confidence intervals). In addition, few of the eligible studies/surveys were recent: 9 out of 11 studies collected data over 7 years ago. Finally, consideration of biases arising from use of BMI-for-age was not carried out (0/11 eligible studies referred to this major source of bias).

**Table 2:** Study quality appraisal summary using the Joanna Briggs Institute, JBI, tool [15]

Criteria-Paper	AlBlooshi, 2016 [18]	AlJunaibi, 2013 [20]	Musaiger, 2012 [19]	Al-Hazzaa, 2014 [21]	Musaiger, 2016 [22]	Al-Awadhi, 2013 [2]	Al-Haifi, 2013 [5]	Alrashidi, 2015 [26]	El-Ghaziri, 2011 [27]	Elkum, 2016 [28]	KNSS,^a^ 2016 [29]
1. Sampling frame appropriate?	No	No	No	No	No	No	No	No	No	No	No
2. Sample appropriate?	Yes	Yes	Yes	Yes	Yes	Yes	Yes	Yes	Yes	Yes	Unclear
3. Sample size adequate?	Unclear	No	Yes	Yes	Yes	Unclear	Unclear	Unclear	Unclear	No	Yes
4. Subjects & settings described?	Yes	Yes	No	Yes	Yes	Yes	Yes	Yes	Yes	Yes	No
5. Analysis conducted to ensure coverage?	No	No	No	No	No	Yes	Unclear	No	No	No	No
6. Valid methods used to define obesity?	Yes	Yes	Yes	Yes	Yes	Yes	Yes	Yes	Yes	Yes	Yes
7. Obesity defined in same way for all subjects?	Yes	Yes	Yes	Yes	Yes	Yes	Yes	Yes	Yes	Yes	Yes
8. Appropriate analysis? (numerator, denominator, %, CI)	No	Yes	No	No	No	Yes	No	No	Yes	No	No
9. Response rate adequate/dealt with?	No	Yes	No	No	No	Yes	No	No	No	No	No
Total/9	4	5	5	5	5	7	4	4	5	4	3

^a^Kuwait Nutrition Surveillance System

### Narrative synthesis by GCC nation

#### UAE

Table 3 summarises the three eligible studies from the UAE [18–20]. None of the three studies used representative samples, though one study [18] was very large (*n* = 44,942), relatively recent (data collection 2013–2015), included both nationals and non-nationals, and included a wide age range (3–18 years). In this study [18] by prevalence of obesity according to the WHO definition exceeded one third of the sample in the secondary school-age participants, and there was clear evidence of increasing prevalence with increasing age. The highest prevalence was recorded for age 11–14 years. In two of the three studies from the UAE [19, 20] obesity prevalence estimates were provided using more than one definition of obesity, and in both cases prevalence was substantially lower using the International Obesity Task Force (IOTF) compared to the alternative definitions (from the US Centers for Disease Control and WHO respectively). In the one study which considered differences in obesity prevalence between the sexes, prevalence was much lower among girls than boys [19] .

**Table 3:** Obesity Prevalence, UAE

Author and Year	Sample Size and age (n)	Data Collection (Years)	Definition of Obesity Used	Obesity Prevalence (%) by Definition	Comments on Obesity Prevalence Estimates
Al Blooshi et al., 2016) [18]	44,942 (Males and females) age 3–18 years	2013–2015	International Obesity Task Force (IOTF), World Health Organization 2007 (WHO), and Centers for Disease Control (CDC)	Prevalence estimates for the nationals only	The sample was apparently not representative of the UAE.
	This study was conducted in two phases; the first phase was in 2013–2014(*n* = 15,532) age 4–12 years.				
					CIs given for prevalence estimates: No
					Biases considered: No
	The second phase was in 2014–2015 (*n* = 29,410) *n* = 27,078 nationals and 2332 non-nationals age 3–18 years .				
					Prevalence higher in boys than girls and generally higher in older than younger individuals
Musaiger et al., 2012) [19]	605 adolescents aged from 15 to 18 years (*n* = 262 males, 243 females)	2010–2011	IOTF and CDC		The sample was apparently not representative of the UAE.
					Biases considered: No
					CIs given for prevalence estimates: No
					Prevalence higher in boys than girls
Al Junaibi et al 2013 [20]	1541, aged 6–19 years (*n* = 1770 males, *n* = 770 females)	January–December 2011	IOTF and CDC		The sample was representative of Abu Dhabi but not the entire UAE.
					CIs given for prevalence estimates: No
					Biases considered: No
					The authors used IOTF to define obesity prevalence but data using IOTF not shown.
					Prevalence higher in older individuals and slightly higher in boys than girls

#### KSA

Table 4 summarises the evidence from the two eligible studies in the KSA [21, 22]. Neither of these studies was based on nationally representative samples. Both studies included adolescents only, and in both studies prevalence of obesity was much higher in boys than girls.

**Table 4:** Obesity Prevalence, Kingdom of Saudi Arabia

Author and year	Sample size (n) and age of sample	Data collection (Years)	Definition of obesity used	Obesity prevalence by methods	Comments on obesity prevalence estimates
Al-Hazzaa et al., 2014) [21]	2908 (*n* = 1401 males, *n* = 1507 females) aged 14 to 19 years	2009–2010	IOTF		The sample was apparently representative of Al-Khobar, Jeddah and Riyadh, but not representative of KSA
					CIs given for prevalence estimates: No
					Biases considered: No
					Prevalence higher in boys than girls
Musaiger et al., 2016) [22]	968 adolescents aged 15–18 years (*n* = 518 males, *n* = 450 females)	2013–2014	IOTF, WHO		The sample represents Dammam city but was apparently not representative of KSA.
					CIs given for prevalence estimates: No
					Biases considered: No
					Prevalence higher in boys than girls

#### Kuwait

Table 5 summarises the 8 eligible studies from Kuwait. A number of the eligible studies were not based on representative samples, were not very recent, and had relatively small samples. The most informative of the data sources from Kuwait was the large and recent nationally representative survey from 2016 [23]. In this survey there was clear evidence of increasing prevalence of obesity with increasing age, and by adolescence one-quarter to one third of participants were obese according to the WHO definition. Among the other 7 eligible studies from Kuwait (Table 5), 3 reported comparisons between prevalence estimates according to the definition of obesity used. Prevalence estimates were generally lower with the IOTF definition than the CDC and WHO BMI-for-age definitions (Table 5). Four studies compared prevalence between the sexes, and in all cases found that prevalence was lower among girls than boys, though prevalence was still high among the girls (typically ranging between 20 and 45% of girls in the samples studied).

**Table 5:** Obesity Prevalence, Kuwait

Author and year	Sample size (n) and age	Data collection (Years)	Definition of obesity used	Prevalence by methods	Comments on obesity prevalence estimates
Al-Haifi et al., 2013) [5]	906 (males *n* = 463, females 443) aged between 14 and 19 years	2009–2010	IOTF, WHO		The sample was from Kuwait city, but apparently not representative of Kuwait
					CIs given: No
					Biases considered: No
					WHO method used for age 18–19 years; but data not included.
					Prevalence slightly higher in boys than girls
El-Ghaziri et al.,(2011) [27]	499 10–14-years (males *n* = 317, girls *n* = 182).	2009	IOTF,WHO, CDC		The sample was from Kuwait city but apparently not representative of Kuwait
					Biases considered: No
					Prevalence differences between the sexes not given
Elkum et al., 2016) [28]	6574 6–18 years (females *n* = 3973, *n* = 2601 males)	2012–2013	IOTF,WHO,CDC		The sample was from Kuwait city apparently not representative of Kuwait
					Biases considered: No
					Prevalence not given for the sexes separately.
Musaiger et al., 2016) [22]	706 aged 15–18 years (*n* = 343 males, *n* = 363females)	2013–2014	WHO, IOTF		The sample was from Kuwait city, apparently not representative of Kuwait
					CIs given: No
					Biases considered: No
					Prevalence higher in boys than girls
Musaiger et al., 2012) [19]	4698 age 15 to 18 years (*n* = 2240 males, *n* = 2458 females).	2010–2011	IOTF, CDC		The sample was apparently representative of Kuwait city, but not representative of Kuwait
					CIs given: Yes
					Biases considered: No
					Prevalence higher in boys than girls.
Alrashidi et al 2015 [26]	960 11-14 years females(*n* = 480) and males (n = 480	February–June 2013	WHO		The sample was from Kuwait city, apparently not representative of Kuwait
					CIs given: No
					Biases considered: No
Al-Awadhi et al 2013 [2]	1273 females, age 15–19 years	2010	CDC	18.3% (95% CI: 16.2–20.6%)	The sample was apparently representative of Kuwait, CIs given: Yes
					Biases considered: No
The Kuwait Nutrition Surveillance system:2016 Annual report,Ministry of Health [29]	12,396, age 5-17 years. (*n* = 6251 males and *n* = 6145 females)	2016	WHO	Overall prevalence of obesity was 25.9%	The sample was representative of Kuwait
					CIs given: No
					Biases considered: No
					Prevalence not given for the sexes separately.

Footnotes: There were no eligible published data or grey literature for 3 of the GCC countries: Bahrain, Oman and Qatar

Where authors acknowledged biases in prevalence estimation with BMI this has been noted. Where authors reported CIs for their prevalence estimates these have been provided in the Tables. *CDC* Centers for Disease Control and Prevention, *BMI* Body mass index, *IOTF* International Obesity Task Force, *WHO* World Health Organisation, *KSA* Kingdom of Saudi Arabia, *UAE* United Arab Emirates

## Discussion

This systematic review showed that evidence on the prevalence of obesity among school age children and adolescents in the GCC states is limited. Only one nationally representative survey was identified, and only 3/6 GCC states had any eligible data from the past 11 years, with multiple gaps in the evidence (e.g. for certain age groups) and weaknesses in the evidence (e.g. reliance on non-representative samples, lack of national surveys). More extensive and higher quality surveillance of obesity among school age children and adolescents in the GCC is required in future if the GCC states are to address the obesity epidemic effectively [7]. Regular high quality surveillance is essential to assess the scale of the obesity problem, to identify trends and inequalities, to drive obesity prevention and control measures, and to assess the impact of policy measures aimed at obesity prevention and control [7].

Despite limitations in the evidence base on obesity prevalence in the GCC nations noted above, some trends were apparent from the 11 eligible studies. First, the prevalence of obesity according to BMI-for-age was very high. For example, prevalence of obesity in UAE according to the WHO definition exceeded one third of the sample in the secondary school-age participants, and increased with increasing age. One-quarter to one third of participants were obese according to the WHO definition in the Kuwaiti national survey. Moreover, BMI-for-age substantially underestimates the prevalence of obesity (excessive fatness) in children [12, 13] so ‘true’ prevalence of obesity in these studies in the GCC would have been even higher if this bias arising from use of the BMI had been accounted for. None of the eligible studies or surveys acknowledged that their prevalence estimates were subject to this source of bias, or attempted to adjust for it. A large recent study [24] across Africa found that the WHO-BMI-for-age definition of obesity only identified around one third of children with excessive body fatness measured by a reference method (Total Body Water). Second, in most of the eligible studies the prevalence of obesity was higher in boys than girls, suggesting that this is a real difference in susceptibility to paediatric obesity in the GCC states. It should be noted that prevalence of obesity among the girls would also be regarded as very high relative to other nations [10, 11]. Third, the eligible studies and surveys which compared prevalence estimates by the different definitions based on BMI-for-age found consistently that prevalence was substantially lower when the IOTF definition of obesity was used compared to definitions based on the CDC or WHO, consistent with previous evidence [12].

There are no previous systematic reviews of the prevalence of child or adolescent obesity from the GCC, and so the results of the present study cannot be compared easily with other evidence. Comparisons of prevalence of obesity among children and adolescents in the GCC with those living in other countries is also difficult because of differences in the timing of the studies, differences in the definitions of obesity used, and whether or not obesity prevalence estimates (as distinct from overweight prevalence estimates, or prevalence of overweight and obesity combined) can be found in published studies.

The present review found major limitations of obesity surveillance in the GCC, notably the apparent lack of any recent surveillance data from 3 of the 6 GCC countries, the availability of nationally representative sample data from only 1/6 GCC countries, the small sample sizes and scarcity of power calculations (and confidence intervals around prevalence estimates), and the fact that bias in the use of BMI-for-age to generate prevalence estimates was not considered by any of the 11 eligible studies/surveys. In addition, eligible study and survey response rates were often very low (under 50%), and not reported in all of the eligible studies and surveys. It should be noted that many of the studies did not set out to obtain nationally representative samples, and estimating obesity prevalence was not a primary aim of all of the eligible studies. In addition, a checklist for guiding/assessing the quality of prevalence studies [15] only became available after many of the eligible studies were conducted. Future studies and surveys of child and adolescent obesity prevalence in the GCC states and elsewhere may find it useful to refer to the checklist for assessment of prevalence study quality used in the present study [15].

This review had a number of strengths. First, it focused on obesity-rather than overweight and obesity. While obesity and overweight are often combined somewhat casually in paediatric prevalence studies they are not equivalent clinically or biologically in children, as in adults: there is currently a very large body of consistent evidence of adverse health effects of obesity in childhood and adolescence [23, 25], but the adverse health impact of overweight in childhood and adolescence is much less clear at present. Second, the present review attempted to provide evidence of most relevance and highest quality, by including only relatively recent studies, and only those which used acceptable objective measures of obesity (rather than self-or parent reports), and by formal appraisal of study quality. The conduct of the present systematic review was also intended to follow best practice, by using the AMSTAR tool as a guide to the process, and reporting of the review followed PRISMA guidance. Finally, by making use of extensive expert contacts in all of the 6 GCC states, the probability that eligible studies and surveys (including grey literature) were not identified by the conventional literature search was reduced.

The present review also had a number of limitations. The number of eligible studies was relatively small due in part to our decision to exclude studies which collected data prior to 2007. The rationale for this is that we included only recent studies to provide up to date information, especially important given likely recent rapid increases in obesity prevalence in the GCC [1–4, 8, 9]. Including older studies would have increased the size of the evidence base, but also made it much less generalisable to contemporary GCC populations. The literature search was limited to English language for practical reasons, but any grey literature or other studies suggested by expert contacts in the GCC published in Arabic would also have been considered if identified. The first author is from Kuwait, which may have biased the grey literature searching towards Kuwaiti sources of evidence. However, author connections in relevant institutions in the rest of the GCC are good, and responses from those contacts were generally informative (Additional file 2). It therefore seems unlikely that useful sources of evidence from the other GCC states, such as recent nationally representative surveys, were missed.

## Conclusions

There is a major gap in the literature on the childhood and adolescent obesity prevalence in the GCC states, with the exception of Kuwait. New research/surveys are needed for those countries in the GCC apparently not doing surveillance of child and adolescent obesity prevalence. For those countries where studies and surveys have been carried out, greater attention could be paid to the quality appraisal issues identified by the present review.

## Additional files

Additional file 1: **Table S1.** Literature search terms in Medline. Search terms and syntax in Medline. (DOCX 14 kb)

Additional file 2: **Table S2.** GCC experts consulted on search findings and missing studies. Summary of experts contacted to check on search results, their affiliations, and their responses. (DOCX 14 kb)

Additional file 3: **Table S3.** List of excluded studies. Summary of full-text screened studies excluded, with reasons for exclusion. (DOCX 26 kb)

## Abbreviations

AMSTAR: Assessment of systematic reviews tool
BMI: Body mass index
CDC: Centers for Disease Control and Prevention
GCC: the Gulf Cooperation Council
IOTF: International Obesity Task Force
JBI: Joanna Briggs Institute
KSA: Kingdom of Saudi Arabia
UAE: United Arab Emirates
WHO: World Health Organization
