# Supplementary material for: Prevalence of obesity among school-age children and adolescents in the Gulf cooperation council (GCC) states: a systematic review
Source: BMC Obes. 2019 Jan 8;6:3. doi: 10.1186/s40608-018-0221-5 (PMC6323696; doi:10.1186/s40608-018-0221-5)
Supplement: Supplementary file 1 — Table S1. Literature search terms in Medline. Search terms and syntax in Medline. (DOCX 14 kb) [file 40608_2018_221_MOESM1_ESM.docx]

Additional file 1: Table S1 Literature search terms in Ovid Medline.

| 1. “Obesity prevalence." 2. “Children” , ‘’Child’’ 3. “Adolescent” or “teen" Or ‘’youth’’ 4. “Gulf Countries” or “Arabian Gulf” 5. “GCC” or “Middle East” 6. “Saudi Arabia”, “Kuwait”, “Oman”, or “Bahrain”, “Qatar”, 7. “United Arab Emirates”, or “UAE” 8. “Reviews”, “Literature”, or “Systematic” 9. #(1)&#(2)#(4). 10. #(1)&#(3)#(5) 11. #(1)&#(2)#(6) 12. #(1)&#(2)#(6). 13. Limit to English language |
| --- |
|  |

The search strategy was replicated in the other databases, but with minor changes of syntax as required by each database.
