# Supplementary material for: Prevalence of obesity among school-age children and adolescents in the Gulf cooperation council (GCC) states: a systematic review
Source: BMC Obes. 2019 Jan 8;6:3. doi: 10.1186/s40608-018-0221-5 (PMC6323696; doi:10.1186/s40608-018-0221-5)
Supplement: Supplementary file 2 — Table S2. GCC experts consulted on search findings and missing studies. Summary of experts contacted to check on search results, their affiliations, and their responses. (DOCX 14 kb) [file 40608_2018_221_MOESM2_ESM.docx]

**Additional file 2: Table S2 GCC experts consulted on search findings and missing studies.**

| **Contact No** | **GCC Country** | **Location** | **Response** |
| --- | --- | --- | --- |
| 1 | Kuwait | Sport Section, Kuwait Ministry of Health | NO |
| 2 | Kuwait | Kuwait University | Yes |
| 3 | Kuwait | Kuwait University | Yes |
| 4 | Kuwait | Ministry of Education | Yes |
| 5 | Kuwait | Public Authority for Food and Nutrition | Yes |
| 6 | Kuwait | Ministry of Education | No |
| 7 | Kuwait | Ministry of Health | Yes |
| 8 | Kuwait | Ministry of Education | Yes |
| 9 | Kuwait | Public Authority for Food and Nutrition | No |
| 10 | Kuwait University | Kuwait University | No |
| 11 | UAE | College of Medicine & Health Science | Yes |
| 12 | UAE | Emirates University | No |
| 13 | UAE | Ministry of Health | Yes |
| 14 | Bahrain | Arabian Gulf University | No |
| 15 | Bahrain | Ministry of Health | Yes |
| 16 | Qatar | Aspetar Sports Medicine Hospital | No |
| 17 | Qatar | Hamad Hospital | No |
| 18 | KSA | Al Majamaa University | Yes |
| 19 | KSA | King Saud University | Yes |
| 20 | KSA | Dammam University | No |
| 21 | Oman | Ministry of Health | Yes |
| 22 | KSA | Ministry of Health | Yes |
| 23 | KSA | Ministry of Health | Yes |
