# Supplementary material for: Prevalence of obesity among school-age children and adolescents in the Gulf cooperation council (GCC) states: a systematic review
Source: BMC Obes. 2019 Jan 8;6:3. doi: 10.1186/s40608-018-0221-5 (PMC6323696; doi:10.1186/s40608-018-0221-5)
Supplement: Supplementary file 3 — Table S3. List of excluded studies. Summary of full-text screened studies excluded, with reasons for exclusion. (DOCX 26 kb) [file 40608_2018_221_MOESM3_ESM.docx]

**Additional file 3: Table S3 List of excluded studies**

| Authors, year | Study title | Reason for exclusion |
| --- | --- | --- |
| Abdul-Rasoul, 2012 | Obesity in children and adolescents in Gulf countries: Facts and solutions. | Old data before 2007 |
| Al-Dossary *et al.*, 2010 | Obesity in Saudi children: a dangerous reality | Old data before 2007 |
| Al-Kilani *et al.*, 2012 | Trends of obesity and overweight among college students in Oman: A cross sectional study | Wrong population group |
| Al-Refaee *et al.*, 2013 | The rising tide of overweight among Kuwaiti children: study from Al-Adan Hospital, Kuwait. | Overweight data, no obesity data |
| Al Shehri, 2013 | Obesity among Saudi children. | Old data before 2017 |
| Bader *et al.*, 2008 | Overweight and obesity among adolescents in Bahrain | Combined overweight and obesity prevalence |
| Boodai *et al.*, 2014a | Prevalence of cardiometabolic risk factors and metabolic syndrome in obese Kuwaiti adolescents | Metabolic syndrome study; No obesity prevalence data |
| Boodai *et al.*, 2014b | National Adolescent Treatment Trial for Obesity in Kuwait (NATTO): project design and results of a randomised controlled trial of a good practice approach to treatment of adolescent obesity in Kuwait. | No relevant obesity prevalence data |
| El-Bayoumy *et al.*, 2009 | Prevalence of Obesity Among Adolescents (10 to 14 Years) in Kuwait. | Old data before 2007 |
| El Mouzan *et al.*, 2010 | Prevalence of overweight and obesity in Saudi children and adolescents | Old data before 2007 |
| Farrag Nesrine S., 2017 | A systematic review of childhood obesity in the Middle East and North Africa (MENA) region: Prevalence and risk factors meta-analysis. | Old data before 2007 |
| Gharib and Rasheed, 2008 | Obesity among Bahrani children and adolescents: Prevalence and associated factors | Old data before 2007 |
| Hammad and Berry, 2016  [3] | The Child Obesity Epidemic in Saudi Arabia: A Review of the Literature. | Old data before 2007 |
| Jackson *et al.*, 2011 | Waist circumference percentiles for Kuwaiti children and adolescents. | No prevalence data |
| Mandeya and Kridli, 2014 | Childhood overweight and obesity in Qatar: A literature review. | Old data before 2007 |
| Mirmiran *et al.*, 2010 | Childhood obesity in the Middle East: a review | Old data before 2007 |
| Musaiger, 2011 | Overweight and obesity in eastern Mediterranean region: prevalence and possible causes. | Old data before 2007 |
| Musaiger *et al.*, 2012 | Body weight perception among adolescents in Dubai, United Arab Emirates. | Old data before 2007 |
| Musaiger and Al-Mannai, 2013 | Role of obesity and media in body weight concern among female university students in Kuwait. | Body Image, No obesity prevalence data |
| Musaiger *et al.*, 2013 | Risk of disordered eating attitudes among adolescents in seven Arab countries by gender and obesity: a cross-cultural study. | Eating Attitudes , No obesity prevalence data |
| Musaiger *et al.*, 2014 | Social, dietary and lifestyle factors associated with obesity among Bahraini adolescents | Old data before 2007 |
| Musaiger *et al.*, 2016 | Disordered Eating Attitudes Among University Students in Kuwait: The Role of Gender and Obesity. | Wrong population group |
| Ng *et al.*, 2014  [6] | Global, regional, and national prevalence of overweight and obesity in children and adults during 1980-2013: a systematic analysis for the Global Burden of Disease Study 2013 | Old data before 2007 |
| Rizk and Yousef, 2012 | Association of lipid profile and waist circumference as cardiovascular risk factors for overweight and obesity among school children in Qatar. | Old data before 2007 |
| Shaban *et al.*, 2017 | Corrigendum to Perceived Body Image, Eating Behavior, and Sedentary Activities and Body Mass Index Categories in Kuwaiti Female Adolescents | Self-image, no obesity prevalence data. |
| Suraya *et al.*, 2017 | Effect of obesity on academic grades among Saudi female medical students at College of Medicine | Wrong population group |
| Zaal *et al.*, 2011 | Anthropometric characteristics and obesity among adolescents in the United Arab Emirates. | No obesity prevalence data |

The updated search in November 2018 identified a further four studies which were full-text screened but ineligible, details as follows: Garemo et al (2018) studied an ineligible population group (Pre-school children); Al Omar et al (2018) used an ineligible means of defining obesity in adolescents (BMI rather than BMI-for-age); Nanhas et al 2018 did not present any prevalence data (protocol paper); Rey-Lopez et al 2018 did not present obesity prevalence data..
